# A review of consecutive cardiac arrests in 2007 and 2012 at a regional hospital in Denmark: a retrospective cohort study

**DOI:** 10.1186/1757-7241-21-S2-A13

**Published:** 2013-09-09

**Authors:** Lisbeth Quitzau, Henriette Ullerup-Aagaard, Mikkel Brabrand

**Affiliations:** 1 Department of Anesthesiology, Sygehus Lillebaelt, Kolding, Denmark; 2 Department of Cardiology, Sydvestjysk Sygehus Esbjerg, Denmark; 3 Department of Emergency Medicine, Sydvestjysk Sygehus Esbjerg, Denmark

## Background

Not much is known about the fate of victims of cardiac arrest (CA) in Denmark. We performed the present study to describe the events surrounding CA at a regional hospital in Denmark.

## Methods

Retrospective analysis of all consecutive CA at Sydvestjysk Sygehus (SVS) in 2007 and 2012, i.e. two years after implementation of new international resuscitation guidelines. The events were identified using a registry in the Department of Anesthesiology. Using a unique personal identification number, we retrieved the patient records and extracted the relevant data.

## Results

We identified 246 cardiac arrests; 154 out-of-hospital and 90 in-hospital (and 2 unknown). 66% were male and the median age was 69 years (range 11-99 years). 38% occurred during daytime, 33% during evenings and 29% at night.

Over all, the primary cause was unknown in 85/246 (29%). There was a decrease in CA caused by acute coronary syndrome (ACS) from 29/117 (25%) in 2007 to 19/129 (15%) in 2012, and an increase caused by respiratory insufficiency from 18/117 (15%) in 2007 to 31/129 (24%) in 2012.

In 2007 83/117 (71%) presented with asystolia/PEA compared to 65/129 (50%) in 2012. The proportion of ventricular tachycardia/fibrillation remained unchanged.

27/117 (23%) achieved return of spontaneous circulation (ROSC) in 2007 and 60/129 (47%) in 2012. 18/60 (30%) were discharged or transferred for further treatment in 2012, in comparison to 14/27 (52%) in 2007.

## Conclusion

Most CA at SVS occurred out-of hospital. The majority were men and the median age was 69 years. They were evenly distributed around the clock. The primary cause was mainly unknown but an increasing number was caused by respiratory insufficiency and a decreasing number by ACS. Asystolia as the presenting rhythm was decreasing. More patients gained ROSC, but the proportion that were discharged or transferred to a university hospital decreased.

